# Evaluative Conditioning with Facial Stimuli in Dementia Patients

**DOI:** 10.1155/2013/854643

**Published:** 2012-09-04

**Authors:** Andreas Blessing, Jacqueline Zöllig, Roland Weierstall, Gerhard Dammann, Mike Martin

**Affiliations:** ^1^Department of Psychology-Gerontopsychology, University of Zürich, Binzmühlestrasse 14/24, 8050 Zürich, Switzerland; ^2^Psychiatric Clinic of Müensterlingen, P.O. Box 154, 8596 Müensterlingen, Switzerland; ^3^Department of Psychology-Clinical Psychology and Neuropsychology, University of Konstanz, P.O. Box 23/25, 78457 Konstanz, Germany

## Abstract

We present results of a study investigating evaluative learning in dementia patients with a classic evaluative conditioning paradigm. Picture pairs of three unfamiliar faces with liked, disliked, or neutral faces, that were rated prior to the presentation, were presented 10 times each to a group of dementia patients (*N* = 15) and healthy controls (*N* = 14) in random order. Valence ratings of all faces were assessed before and after presentation. In contrast to controls, dementia patients changed their valence ratings of unfamiliar faces according to their pairing with either a liked or disliked face, although they were not able to explicitly assign the picture pairs after the presentation. Our finding suggests preserved evaluative conditioning in dementia patients. However, the result has to be considered preliminary, as it is unclear which factors prevented the predicted rating changes in the expected direction in the control group.

## 1. Introduction

The majority of our likes and dislikes we acquire throughout the lifespan are the product of learning [1]. One of the most important ways through which stimuli acquire affective meaning is the change of valence that results from pairing one stimulus (CS) with a positive or negative affective stimulus (UCS). As a result, the CS acquires a valence congruent with the affective value of the UCS. This effect is called (associative) evaluative conditioning and has been demonstrated in humans with a large variety of procedures and stimuli (e.g., [2–4]; for a meta-analysis see [5]). 

In dementia severely impaired explicit memory is the core feature. Nonetheless, patients with dementia might still be able to implicitly learn affective reactions through the process of evaluative conditioning. However, to the best of our knowledge, no study applied this approach in dementia patients. Another classical paradigm that has already been used to investigate conditioning of affective reactions in this population is fear conditioning. Indeed, two studies indicate that fear conditioning is impaired in dementia patients [6, 7]. Even though fear conditioning is impaired, evaluative conditioning might still be possible in dementia patients. Some researchers have argued that while on a procedural level evaluative conditioning is similar to fear conditioning, the underlying processes might be different. It was hypothesized that fear conditioning is an instance of signal learning; it is learned that the UCS is going to appear after the presentation of the CS [8]. Evaluative conditioning, on the other hand, only involves a reference to the UCS without expectation of its occurrence [9]. Thus, explicit knowledge about the CS-UCS relation seems crucial in fear conditioning while evaluative conditioning can be demonstrated in the absence of contingency awareness [10–12]. Since there could be variables that play a different role in these forms of learning; dementia patients might show intact evaluative conditioning despite impaired fear conditioning. 

Indeed, some studies indicate that dementia patients might retain the capacity to acquire affective reactions [13, 14]. Blessing et al. [13] demonstrated that dementia patients' affective reactions can be influenced by pairing faces with fictional biographical content that characterized the depicted persons in terms of either positive or negative traits. Pictures were rated before and at two different time points after the presentation of fictional biographical content with respect to valence and arousal. Recognition of pictures and free recall of fictional biographical content was tested. Patients changed their ratings of pictures according to the biographical information presented, but did not recognize pictures above chance level or recall biographical information. These findings were replicated and extended in a subsequent study [14]. The paradigm used by Blessing et al. [13, 14] seems similar to standard evaluative conditioning paradigms. However, there are two main differences: (1) in standard evaluative conditioning paradigms the subjects are not explicitly informed about the interrelation between stimuli in the learning phase. In the paradigm used by Blessing et al. [13, 14] the paring of the CS and UCS is made explicit and thus, the procedure cannot be described as a simple cooccurrence of stimuli; (2) in contrast to the approach of Blessing et al. [13, 14] the UCS and CS in evaluative conditioning paradigms belong to the same type of stimuli (e.g., faces).

Hence, in the present study we addressed the question if affective evaluations of dementia patients can be manipulated using a standard evaluative conditioning paradigm. 

## 2. Design and Methods

*Participants*. Demographical data and clinical characteristics of both groups are listed in Table 1. The study included 15 dementia patients (diagnosed as Alzheimer's disease (*N* = 10) or mixed dementia (*N* = 5)). Patients were outpatients in the Memory Clinic of the Psychiatric Clinic of Muensterlingen at the time of testing. All patients were diagnosed by a multidisciplinary team of the hospital ward using ICD 10 criteria [15]. The diagnosis was based on general medical, neurological, and neuropsychological examinations. All patients had received medical attendance including magnetic resonance imaging and specific screening blood tests, in order to exclude syphilis, diabetes, thyroid disorders, and vitamin B12 and folic acid deficiency. 

Fourteen healthy age-matched participants were recruited as controls. Controls were noninstitutionalized and managed their own household. They reported that they had no known CNS diseases, contact with toxic substances, or substance abuse. All participants gave written informed consent. The study protocol was approved by the local ethics review board.

*Pictures*. Test stimuli were 10 pictures of neutral (i.e., displaying no facial expression of emotion) unfamiliar faces (6 female: 3 young, 3 old; 4 male: 2 young, 2 old) and one picture of a happy young, female adult as well as one picture of a happy old, male adult selected from the Productive Aging Laboratory Face database [16]. However, based on affective valence ratings of these pictures in other studies (dann hier Referenz), some of the included faces were also rated as positive or negative. The happy faces were added to increase the variance in subjective liking between pictures.

*Emotional Ratings*. Valence ratings were obtained via a Self-Assessment Manikin (SAM; [17]). The SAM was designed to assess subjective ratings of participants' emotional responses and minimize the influence of language and culture on ratings. The SAM valence rating scale has been successfully used in previous studies with dementia patients [13, 14]. Using the paper-pencil version of this instrument, participants rated the stimuli as to their emotional valence (range: 1–9). 

### 2.1. Procedure

Similar to other studies investigating evaluative conditioning we used a cover story in order to minimize demand effects. Participants were told that the aim of the experiment was to examine the relationship between mood and subjective affective evaluation. In line with this cover story participants rated their mood on a five-point Likert scale (very good/good/normal/bad/very bad). Subsequently, the pictures were presented to participants one after the other and rated with respect to valence using the SAM rating scale. The experimenter emphasized to rely on the first, spontaneous reaction towards the stimuli. Pictures were presented in four different pseudorandom sequences. After the valence rating an individual, unequivocal preference order of all stimuli was established. The pictures that received identical valence ratings were again presented to participants who then had to decide which of the pictures they preferred and the respective picture was put aside. Again, the participants had to choose which of the remaining pictures with identical valence ratings they preferred and so on. 

The most preferred stimulus out of the 12 faces was used as the liked (L) stimulus and the stimulus with the lowest ranking was used as the disliked (D) stimulus. The four stimuli ranked 5th, 6th, 7th, and 8th were used as neutral (N) stimuli. The experimenter entered these six individual L, D, and N stimuli in a computer program (presentations 11.3) that automatically formed three stimuli pairs (i.e., Neutral-Liked, Neutral-Disliked, and Neutral-Neutral) by randomly assigning the neutral stimuli. Participants were then seated about 50 cm from the computer screen and instructed to look at the pictures that would appear on the screen. Participants were asked to refrain from talking whenever possible. Each picture from the different pairs (i.e., (N-L), (N-D) or (N-N)) was presented 10 times in the center of the computer screen. A different random sequence was used for each participant. The duration of each stimulus presentation was 1 second and the interstimulus interval (ISI; i.e., onset of the first stimulus of a pair to onset of the second stimulus of a pair) was 4 seconds. The inter-trial interval (ITI; i.e., onset of the first stimulus of the previous trial to onset of the first stimulus of the next trial) was 13 seconds. After the presentation of stimuli on the computer screen, participants were again asked to rate the N, D, and L stimuli on the SAM valence rating scale. The pictures were presented in a random sequence. 

To measure explicit contingency awareness a cued-recall approach was used. The three relevant N stimuli (i.e., the first stimulus of each pair) were placed one after the other in front of participants together with the three stimuli second in the pairs (L, D, N). Participants were asked to indicate which of these three stimuli followed the currently presented N stimulus. The experimenter registered the answer on a response sheet. 

### 2.2. Data Analysis

A repeated measures Analyses of Variance (ANOVAs) was conducted for the valence ratings of the relevant N stimuli (i.e., the first stimulus of each pair). A separate analysis was performed for ratings of stimuli paired with L and D pictures. Type of stimulus pair and measurement point (i.e., valence rating pre and post presentation) were used as within-participant factors and group was included as between subject factor. In the case of significant group differences a separate analysis was performed for both groups. Probabilities of contingency awareness scores in both groups were calculated using the binominal distribution.

## 3. Results

### 3.1. Valence Ratings

The repeated measures ANOVA revealed no main effect of type of stimulus pair (*F*(2,26) = 2.42; *P* > .109) but an interaction between type of stimulus pair and time (*F*(2,26) = 7.354; *P* > .003; see Table 2). This interaction was due to the fact that there was no influence of the type of stimulus pair at baseline (*F*(2,27) = 0.296; *P* > .746), but a significant effect after conditioning in the direction of the experimental manipulation (*F*(2,27) = 5.460; *P* < .010). No interaction between type of stimulus pair and group (*F*(2,26) = 3.023; *P* > .066) or stimulus pair, time, and group (*F*(2,26) = 2.798; *P* > .079) appeared. ANOVA indicated no main effect of time (*F*(1,27) = 0.78; *P* > .385). 

As expected, we found a grater rating change over time for stimuli paired with L and D pictures than stimuli paired with N pictures (see Table 2). We conducted a separate analysis using only pictures paired with L and D pictures to further investigate the influence of the experimental manipulation. The repeated measures ANOVA revealed a main effect of type of stimulus pair (*F*(1,27) = 4962; *P* > .034) along with an interaction between type of stimulus pair and time (*F*(1,26) = 15.237; *P* > .001; see Table 2). We found no significant interaction between type of stimulus pair and group (*F*(2,26) = 2.894; *P* > .10) but a significant interactions between stimulus pair, time, and group (*F*(1,26) = 5.784; *P* < .023). ANOVA indicated no main effect of time (*F*(1,27) = 0.296; *P* > .591). 

Because of the significant interaction between stimulus pair, time, and group, indicating group differences, we performed a separate analysis for each group. The repeated measures ANOVA for valence ratings of pictures paired with L and D stimuli of dementia patients revealed a significant main effect of type of stimulus pair (*F*(1,14) = 16.000; *P* > .001) along with an interaction between type of stimulus pair and time (*F*(1,14) = 29.007; *P* > .001). We found no main effect of time (*F*(1,14) = 0.121; *P* > .733). 

The repeated measures ANOVA for ratings of controls indicated no main effect of type of stimulus pair (*F*(1,13) = .087; *P* > .773) and no interaction between type of stimulus pair and time (*F*(1,13) = .832; *P* > .378). Again, ANOVA indicated no main effect of time (*F*(1,13) = 0.188; *P* > .671). 

### 3.2. Contingency Awareness

In the group of dementia patients, 24.4% of the relevant N stimuli were assigned to the correct stimulus second in the pairs in the forced choice test, which did not differ from chance level (i.e., 33.3%; *P* > .14). In the control group, 31% relevant N stimuli were assigned to the correct stimulus second in the pairs in the forced choice test, which did not differ from chance level (i.e., 33.3%; *P* > .37).

## 4. Discussion

The main aim of the present study was to investigate if affective evaluations of dementia patients can specifically be influenced through a standard evaluative conditioning paradigm. As hypothesized, dementia patients changed their valence ratings of unfamiliar and previously neutral faces according to its pairing with either a liked or disliked face stimulus. Generally, the neutral pictures that were paired with a liked stimulus were rated higher and the neutral picture that was paired with the disliked stimulus was rated lower on the valence dimension after our evaluative conditioning intervention by dementia patients. Thus, results indicate that ratings of initially neutral stimuli can be influenced in the according direction through simple time-near presentation (i.e., pairing) with both a liked as well as a disliked stimuli. It does not seem surprising that our forced choice recognition test indicated no contingency awareness as this is based on the functionality of explicit memory that is severely impaired in dementia patients. 

However, dementia patients significantly differed from controls in the effect of the experimental manipulation. Interestingly, we found no influence of the experimental manipulation in the control group. The negative finding in the control group could indicate that our paradigm did not produce evaluative conditioning effects. Consequently, the detected influence in the AD group could be accounted for by demand effects or other nonevaluative conditioning effects. Another explanation could be that there was only a small effect in the control group that could not be detected due to small sample size. However, the sample size was only slightly smaller than that of the AD group. We looked at the data on the level of individual subjects and found that 11 of 14 subjects in the control group changed their ratings in the expected direction or showed no rating change. The negative result in the control group was due to two participants who showed a strong rating change in the opposite direction. Post hoc analysis indicated a significant influence of the experimental manipulation in the control group after exclusion of these two outliers (*P* < .028). Thus, it seems possible that evaluative conditioning effects in the control group were masked by the influence of outliers in our small sample. The rating changes of outliers could have been motivated by reactance, as observed in other studies investigating evaluative conditioning [18].

Our result could also indicate that evaluative conditioning effects are stronger in dementia patients than in healthy elderly controls. A possible reason for stronger effects could be lower attentional resources in dementia patients due to disease-related cognitive impairments. Some studies indicate that attentional resources have a negative impact on affective learning through evaluative conditioning (e.g. [19, 20]) and accordingly it seems possible that dementia patients show stronger effects. However, there are conflicting findings concerning the influence of attentional resources on evaluative conditioning (e.g., [21]). 

The observed positive change of the valence rating in the neutral stimulus that was paired with another neutral stimulus could be a result of the mere exposure effect. This effect describes the preference for stimuli that have been previously presented over novel stimuli [22]. Preference changes due to the mere exposure effect have also been demonstrated in dementia patients [23–25]. In line with this explanation are findings from other studies investigating evaluative conditioning that report a similar trend of positive changes in evaluations of neutral pictures paired with other neutral pictures over time [26]. 

The results suggest that dementia patients changed their ratings according to the experimental manipulation without contingency awareness, since pairings were not identified above chance level in the forced choice recognition test. In fact, participants of the control group performed better than dementia patients in the recognition test, yet their results did not differ from chance level as well. The results of dementia patients are in line with findings demonstrating evaluative conditioning in healthy participants using subliminally presented stimuli [27–29]. Moreover, Baeyens et al. [26] could show that the individual amount of the evaluative conditioning effect is not related to the number of pairings (i.e., neutral stimulus-affective stimulus) participants were aware of. Furthermore, some results indicate a negative effect of contingency awareness [20]. On the other hand, findings of a recent meta analysis suggest that contingency awareness is an important moderator in evaluative conditioning [5]. 

However, a critical limitation of the present study is our assessment of contingency awareness using a forced choice recognition test. Following the recent discussion of Gawronski and Walther [30], it seems possible that we measured contingency memory rather than contingency awareness. Subjects may have realised the respective pairings during conditioning but may not have been able to remember these pairings explicitly when the recognition test was applied. This may have been the case especially in dementia patients because of severe memory deficits. In addition, our recognition test could have been insensitive to capture postconditioning memory. Subjects may have remembered statistical probabilities that could not be assessed using a forced choice test. Thus, it is also possible that contingency awareness differed between dementia patients and controls in our study and that contingency awareness influenced the results. To prohibit contingency awareness, very long time intervals on the ISI and ITI level were used in the present study. Short ISI could facilitate the detection of contingencies in the combinations of pictures and would enhance contingency awareness. 

Our finding suggests preserved evaluative conditioning in dementia patients. However, this result has to be considered preliminary, since it is unclear what prevented rating changes in the expected direction in the control group in our paradigm. Nevertheless, the results seem to be in contrast to previously reported impaired fear conditioning. As discussed above it is still unresolved whether evaluative conditioning is a form of pavlovian conditioning or if there are variables that are unique to evaluative conditioning. Thus, the results of our study support the notion that different processes are involved in evaluative conditioning and fear conditioning. A relevant difference could be the dependence of pavlovian conditioning on contingency awareness in contrast to evaluative conditioning as discussed above. Another reason for conflicting findings in fear conditioning versus evaluative conditioning studies could be the use of different dependent measures. Fear conditioning studies primarily use skin conductance as a dependent measure, whereas evaluative conditioning studies rely on behavioural measures. This explanation is supported by recent findings from our lab revealing that changes of affective evaluations were not related to skin conductance responses but to heart rate response in dementia patients in a face-emotion association paradigm [31]. Accordingly, there might be a specific impairment in dementia patients with respect to skin conductance response. 

On a neurophysiological level, the underpinnings of evaluative conditioning are not well understood. Some studies indicate that temporal regions and specifically the amygdala are involved in both fear conditioning [32–35] and evaluative conditioning [36]. Accordingly, a similar performance in both types of learning could be expected. However, there is also evidence suggesting that the functionality of the amygdaloid nuclear complex may not be crucial for the occurrence of evaluative conditioning [37]. Hence, preserved evaluative conditioning in dementia patients would be in line with findings demonstrating that despite of impaired fear conditioning evaluative conditioning is preserved in persons with unilateral damage to the amygdaloid nuclear complex [37]. Similarly, Tranel and Damasio [38] report the case of a patient with bilateral damage to the entire medial lobe who could learn connections between unfamiliar persons and affective valence they displayed, despite severely impaired explicit memory. 

In summary, results of our study suggest that dementia patient' affective evaluations of neutral stimuli can be changed through pairing with liked or disliked stimuli. However, caution is warranted since it is not unambiguous what caused these rating changes in our study. Future research should focus on preserved learning processes in dementia patients since they are of great importance for nonpharmacological therapeutic interventions.

## Figures and Tables

**Table 1:** Demographical parameters and clinical characteristics in the study groups.

	Dementia patients	Controls	*P*
*N*	15	14	
Gender (male/female)	11/4	10/4	
Age (years; *M* (SD))	78.7 (6.0)	78.9 (6.5)	>.935
Education (years; *M* (SD))	9.9 (2.1)	11.0 (2.4)	>.207
MMS (*M* (SD))	23.3 (3.1)	28.9 (0.7)	<.001∗∗

Note. *M*: mean, SD: standard deviation.

**Table 2:** Mean valence ratings.

Type of stimulus pair	Time	
	Pre-conditioning *M* (SD)	Post-conditioning *M* (SD)
Neutral-Liked		
Dementia patients Controls	4.93 (0.96) 5.43 (1.34)	6.63 (1.49) 5.86 (1.70)
Neutral-Neutral		
Dementia patients Controls	5.13 (0.34) 5.07 (1.49)	5.60 (0.43) 5.21 (1.57)
Neutral-Disliked		
Dementia patients Controls	5.07 (1.28) 5.57 (1.22)	4.00 (1.69) 5.43 (1.82)

Note. Higher ratings denote higher pleasure. *M*: mean, SD: standard deviation.
